# Recurrent Pancreatic Pseudocysts Due to Alcohol-Related Chronic Pancreatitis With Double-Duct Sign and Spontaneous Rupture

**DOI:** 10.7759/cureus.16039

**Published:** 2021-06-29

**Authors:** Tanveer Hasan, Pranav Jha, Sunil Thippeswamy

**Affiliations:** 1 Internal Medicine, East Kent Hospitals University NHS Foundation Trust, Ashford, GBR; 2 General Medicine, Croydon University Hospital, London, GBR

**Keywords:** pancreatic pseudocysts, double duct sign, chronic pancreatitis, spontaneous rupture, endoscopy ercp, magnetic resonance cholangiopancreatography (mrcp)

## Abstract

A 36-year-old female presented with recurrent attacks of alcohol-related acute on chronic pancreatitis complicated by a pancreatic pseudocyst in the head of the pancreas. The cyst was causing obstruction of the common bile duct (CBD) and pancreatic duct (PD) on magnetic resonance cholangiopancreatography (MRCP). She underwent endoscopic ultrasound (EUS)-guided aspiration of the cyst to dryness. A few months later, she presented with epigastric pain and jaundice. MRI pancreas and MRCP revealed a pancreatic cyst in the head of the pancreas, which had grown in size, compressing the CBD and PD with extra and intrahepatic biliary dilatation. There was a recurrence of a new lobulated peripancreatic pseudocyst, which had ruptured resulting in a large collection compressing the right renal capsule. Percutaneous drainage of the ruptured pseudocyst was performed, followed by endoscopic retrograde cholangiopancreatography (ERCP) that revealed distal CBD stricture, which was stented. Her symptoms improved and she was discharged with no further recurrences of obstructive jaundice during the one-year follow-up period.

## Introduction

Pancreatic pseudocysts are collections of fluid-containing pancreatic enzymes, surrounded by fibrous tissue, which commonly occur as a sequela of pancreatitis [1]. A third of these cysts occur in the head of the pancreas and the rest in the tail. Recurrence of pancreatic pseudocysts after treatment is not uncommon, especially related to alcohol consumption [1,2]. The management often requires more than one intervention, ranging from less invasive percutaneous drainage to more invasive surgery. In this report, we describe a complex and rare case of recurrent pancreatic pseudocysts, which was complicated by spontaneous rupture and double-duct obstruction, thereby requiring multiple interventions.

## Case presentation

A 36-year-old female patient initially presented with recurrent attacks of upper abdominal pain, having been diagnosed with alcohol-related chronic pancreatitis. She had a past medical history of depression, pulmonary embolism, portal vein thrombosis, sickle cell trait, and pituitary microprolactinoma. Her medications included furosemide, pregabalin, methadone, omeprazole, pancreatin, thiamine, and vitamin B. She had 15 pack-years of smoking history and consumed about 42 units of alcohol per week. She was also an intravenous (IV) drug abuser. On examination, she had epigastric tenderness with marked ascites. There were no signs of jaundice or stigmata of chronic liver disease.

Liver function tests were remarkable for raised alkaline phosphatase (ALP) of 337 U/L (normal range: 30-130) and mildly raised alanine aminotransferase (ALT) of 96 U/L (normal range: 0-50). Notably, total bilirubin, albumin, amylase, C-reactive protein (CRP), renal function test, and full blood count were normal. Tumor markers including carcinoembryonic antigen (CEA) and cancer antigen 19-9 (CA 19-9) were within normal limits. Fecal elastase level was 23 ug/g (normal range: >200), suggestive of severe pancreatic insufficiency. HbA1C was 111 mmol/mol (normal range: <42), suggesting type 3C diabetes mellitus.

**Figure 1:**
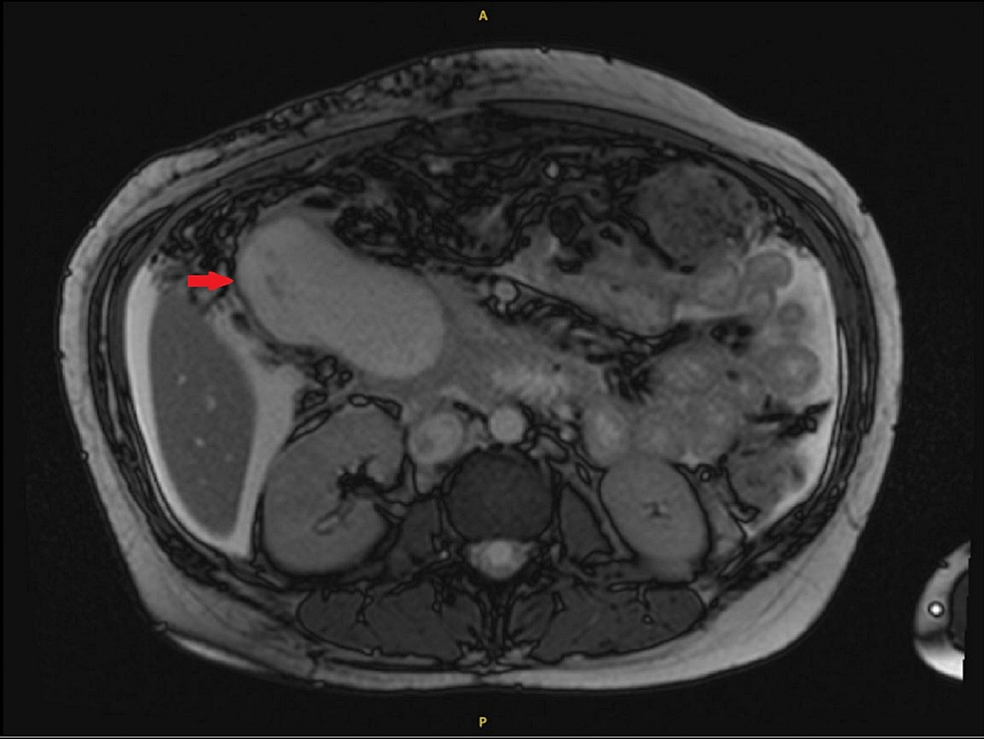
MRI of the pancreas and abdomen on the first visit The image shows a 8 x 3 x 3-cm pseudocyst in the head of the pancreas (red arrow) MRI: magnetic resonance imaging

CT scan of the abdomen and pelvis with contrast revealed features of chronic pancreatitis along with a 7 x 4.5-cm fluid collection inferior to the uncinate process of the pancreas with intra and extrahepatic biliary dilatation. MRI of the pancreas and abdomen confirmed findings of 8 x 6 x 6-cm pseudocyst in the pancreatic head causing mass effect (Figure 1), leading to marked dilatation of the pancreatic duct (PD) at about 6 mm, prominent common bile duct (CBD) of about 11 mm with dilatation of the intrahepatic biliary tree. Moderate ascites was also seen.

100 ml of blood-stained, straw-colored ascitic fluid was obtained under ultrasound guidance. Fluid was sent for cytology and microbiology. Microscopy showed leucocytes of 472/mm^3^, lymphocytes of 5%, and neutrophils of 95%. There was no bacterial growth on culture. serum-ascites albumin gradient (SAAG) was 5 and ascitic fluid amylase was 24 U/L, suggesting reactionary ascites that was infected. Cytology of the fluid revealed fluid predominated by neutrophilic polymorphs and reactive lymphoid elements. The patient was treated conservatively with intravenous antibiotics for infected ascitic fluid and was also started on subcutaneous insulin and oral metformin for glucose control. Her symptoms improved on conservative management, and hence she was discharged with outpatient follow-up.

**Figure 2:**
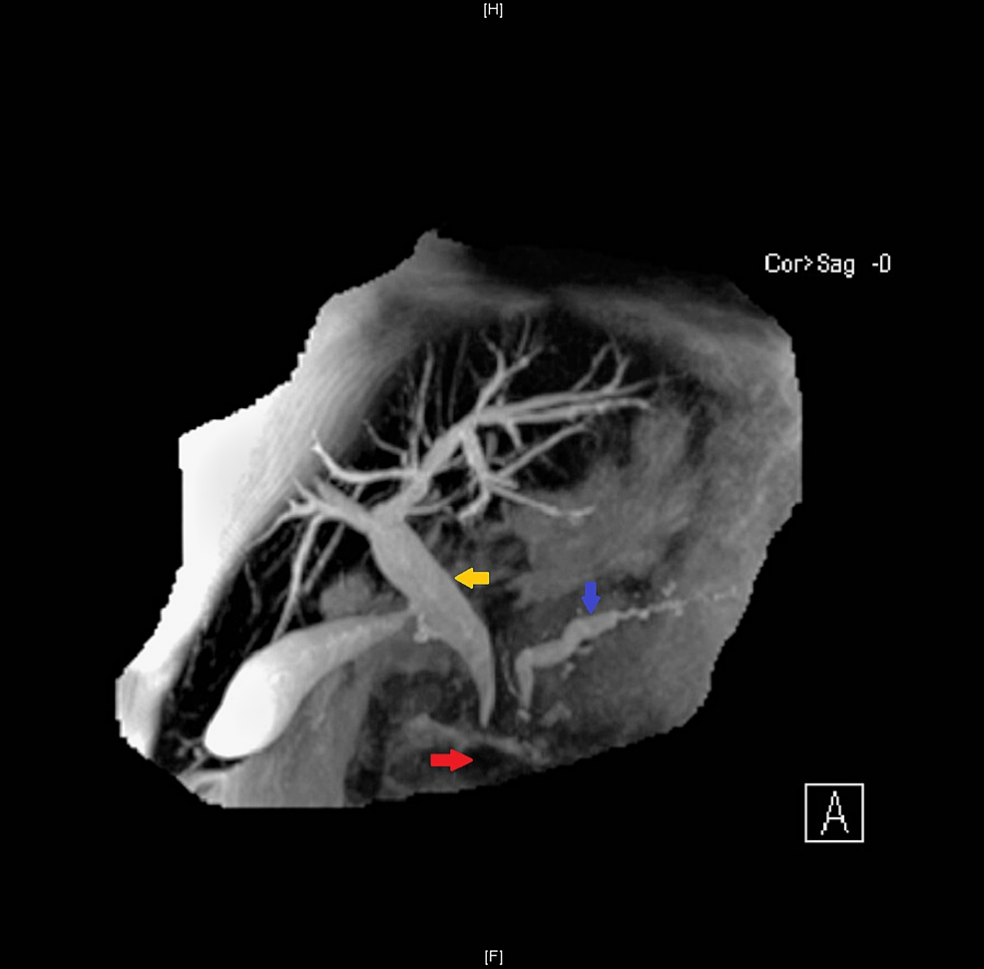
MRCP showing pseudocyst in the head of the pancreas (red arrow) The pseudocyst can be seen exerting mass effect over adjacent structures, causing dilatation of the pancreatic duct (blue arrow) and common bile duct (yellow arrow) - the double-duct sign, with early intrahepatic biliary tree dilatation MRCP: magnetic resonance cholangiopancreatography

She re-presented after a couple of months with obstructive jaundice (markedly raised bilirubin and ALP). On this occasion, magnetic resonance cholangiopancreatography (MRCP) revealed dilatation of the intrahepatic biliary tree, dilatation of the CBD to 13 mm, and dilated PD up to 7 mm secondary to extrinsic obstruction at the level of the pseudocyst in the head of the pancreas (Figure 2).

Endoscopic ultrasound (EUS) was performed and showed a 2.6-cm thick-walled cyst in the head of the pancreas with a 1.5-cm soft tissue nodule in the cyst wall. There was freely floating organized material within the cyst. No locoregional lymph nodes were seen. The coeliac axis and portal confluence were normal. The CBD and main PD were dilated by the cyst. There were no major EUS signs of chronic pancreatitis based on Rosemont criteria. The cyst was aspirated to dryness by EUS guidance with a 20-gauge EchoTip ProCore® needle (Cook Medical, Bloomington, IN). Hemorrhagic, non-mucinous fluid was obtained for chemistry and cytology. The soft tissue nodule was sampled with a 22 GF Acquire^TM^ fine-needle biopsy device (Boston Scientific, Marlborough, MA). The findings from EUS, fluid biochemistry, and the biopsy of cyst nodules were consistent with a pseudocyst. There was no evidence of malignancy or infection. After EUS-guided aspiration of the cyst, her jaundice improved with a significant fall in bilirubin and ALP levels.

After six months, the patient presented with pruritus, worsening abdominal pain, abdominal distension, jaundice, pale stools, and dark urine. She had still been drinking about 30 units of alcohol per week. Examination revealed icterus and uniformly distended abdomen with epigastric tenderness, no guarding or rigidity, and normal bowel sounds.

Her full blood count, renal function, and electrolytes were within normal limits. CRP was mildly elevated at 34 mg/L (0-10). Liver function profile showed albumin of 32 g/L (35-50), protein of 72 g/L (60-80), total bilirubin of 128 µmol/L (0-22), ALP of 844 U/L (30-130), ALT of 53 U/L (0-50) with normal prothrombin time (PT) and activated partial thromboplastin time (aPTT). Renal function and electrolytes were within normal limits.

**Figure 3:**
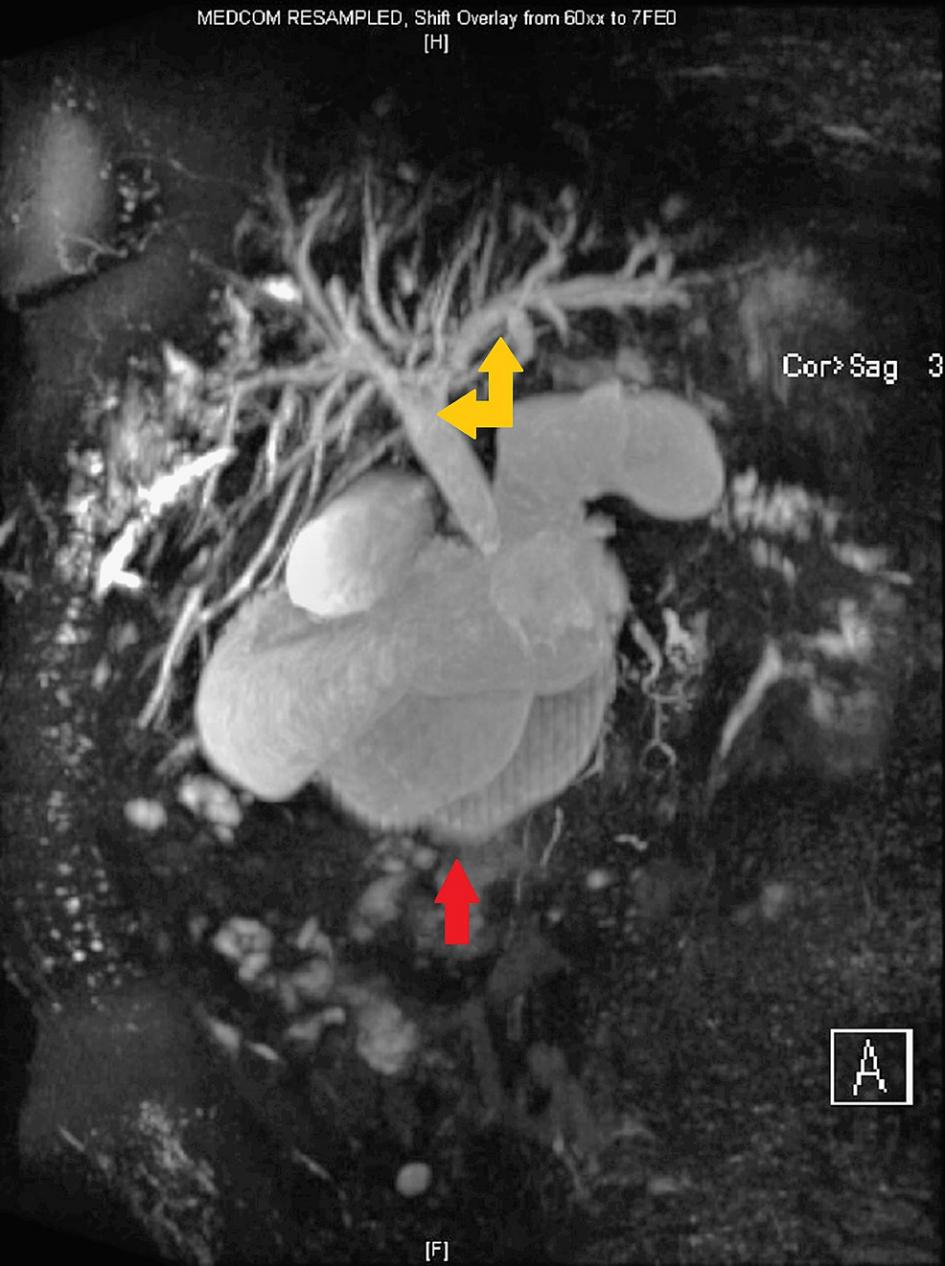
MRCP showing pancreatic pseudocyst in the head of the pancreas (red arrow) The pseudocyst is seen measuring 4.8 x 4.6 cm causing local pressure effect with marked intra and extrahepatic biliary dilatation (yellow arrows) MRCP: magnetic resonance cholangiopancreatography

**Figure 4:**
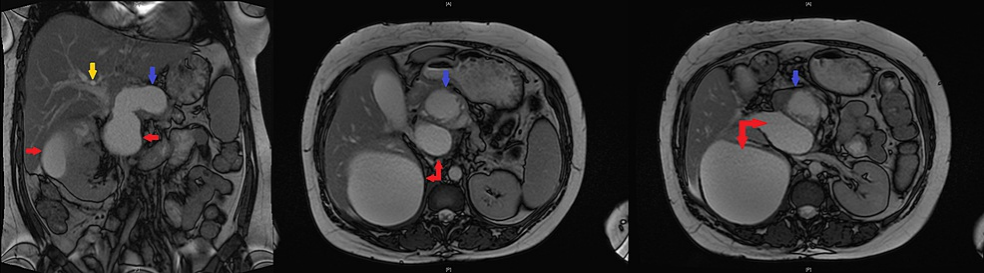
MRI of the pancreas and abdomen The images show evidence of chronic pancreatitis and two pancreatic pseudocysts, one of which is in the head of the pancreas (blue arrows) measuring 4.8 x 4.6 cm causing a local pressure effect on the CBD (yellow arrow). The other large peripancreatic pseudocyst (red arrows) measuring about 7 x 4 cm has ruptured causing collection sized 12.9 x 11.2 x 13.0 cm, compressing on the right renal capsule MRI: magnetic resonance imaging; CBD: common bile duct

CT abdomen suggested recurrence of the pseudocyst in the pancreatic head, causing compression of the CBD and PD leading to dilatation of upstream PD, CBD, and intrahepatic biliary ducts. There was a further large cyst measuring 6.6 x 3.7 cm posterosuperior to the pancreas along with a larger right renal cyst. MRI confirmed evidence of chronic pancreatitis and two pancreatic pseudocysts. One of the cysts in the head of the pancreas measuring 4.8 x 4.6 cm was causing local pressure effect on the CBD, and there was intra and extrahepatic biliary dilatation with the CBD measuring up to 15 mm (previously 13 mm) (Figure 3). The cyst was also causing compression of the pancreatic duct, which was dilated to approximately 7 mm. The other large peripancreatic cyst measuring about 7 x 4 cm located posterosuperior to the body of pancreas had ruptured causing collection sized 12.9 x 11.2 x 13.0 cm, compressing on the right renal capsule (Figure 4).

Ultrasonography-guided percutaneous drainage of the cyst was performed. The fluid obtained from the drainage was turbid and bloody with no growth of microorganisms on culture and negative for malignant cells on cytology. Following the drainage, an endoscopic retrograde cholangiopancreatography (ERCP) was performed that showed a distal CBD stricture, which was stented using a fully covered self-expandable metal stent. Brushings retrieved during ERCP did not show any evidence of malignancy.

Outcome and follow-up

**Figure 5:**
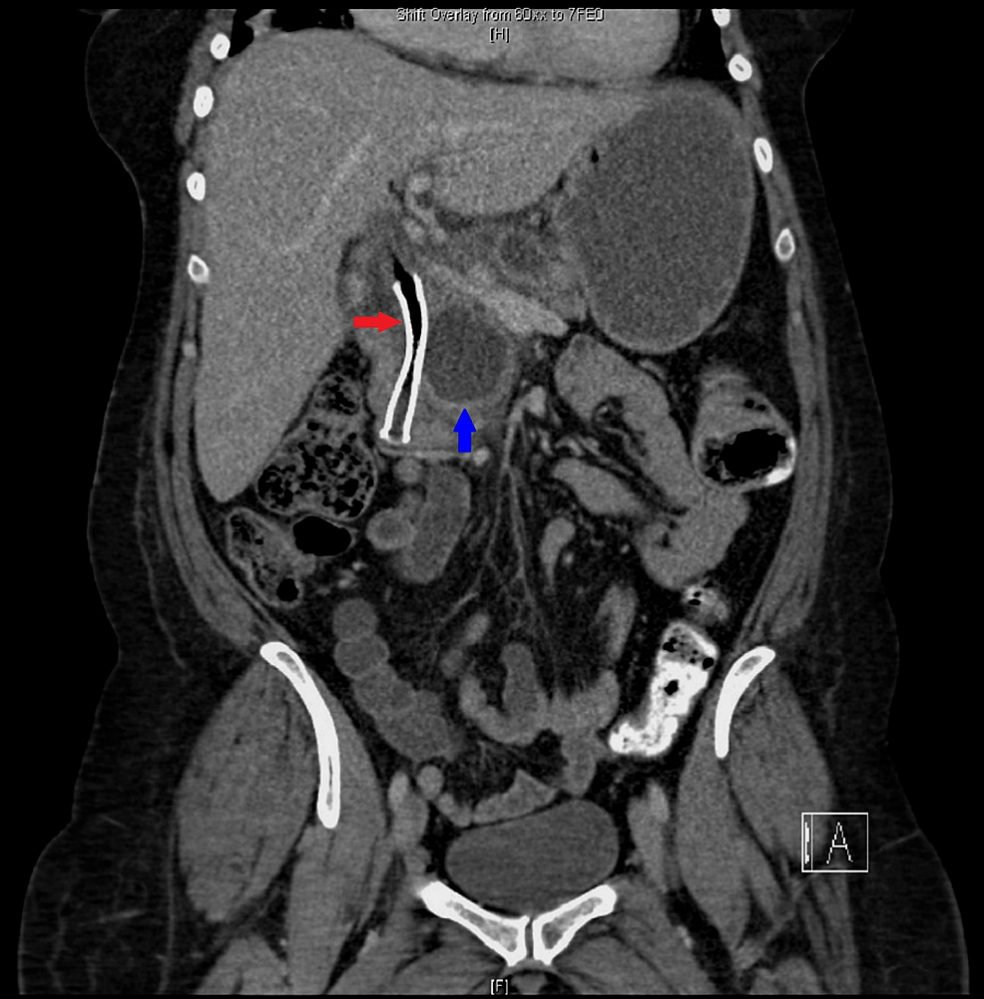
CT abdomen showing multiple pseudocysts (blue arrow), which have significantly reduced in size. A metallic biliary stent is seen, which is patent (red arrow) CT: computed tomography

Following the CBD stent insertion and the percutaneous drainage of the ruptured pseudocyst, her symptoms markedly improved. ALP and total bilirubin levels returned to normal and she was discharged with follow-up in gastroenterology outpatient clinic. She remained well for a while before she re-presented in a couple of months with severe right loin pain and nausea. On this admission, she was febrile with a CRP of 291 mg/L (1-10) and a white blood cell count of 16.9 x 10^9^/l (4-11). CT abdomen was suggestive of infected residual collection at the site of the previous drainage with a significant reduction in the size of the pseudocysts and preserved patency of the metallic biliary stent (Figure 5). She responded well to IV co-amoxiclav and was discharged from the hospital. The patient remained well thereafter, with no further recurrences of obstructive jaundice during the one-year follow-up. Stent removal was planned in a few months but was delayed due to the current coronavirus disease 2019 (COVID-19) pandemic.

## Discussion

After an episode of acute pancreatitis, fluid collections may occur in the peripancreatic tissues in a few weeks to form pancreatic pseudocyst [3]. During pancreatitis, the major PD or one of the ductal branches can get disrupted due to necrosis, resulting in the accumulation of pancreatic secretions mainly consisting of pancreatic enzymes. While most collections undergo spontaneous resolution, in some cases they persist, eventually forming a fibrous wall around the fluid. They are called pseudocysts as they lack a true epithelial lining, unlike true cysts. Pancreatic pseudocyst may be acute or chronic, depending on whether the process that led to the pseudocyst was acute or chronic pancreatitis, respectively. Acute pseudocysts require at least four weeks to develop and are devoid of significant solid debris. The incidence of pancreatic pseudocysts ranges between 20 and 40% in chronic pancreatitis, most notably when related to alcohol consumption [4].

Patients with pancreatic pseudocysts commonly present with abdominal pain and nausea. The cysts can get infected leading to fever. Furthermore, jaundice can occur in pancreatic pseudocyst secondary to compression and narrowing of the terminal CBD due to fibrosis [2]. On rare occasions, pseudocysts can rupture. While rupture into the gastrointestinal tract has a favorable outcome, rupture into the peritoneal cavity and vascular system has a poorer prognosis [5].

The initial diagnostic modality of choice for pancreatic pseudocyst is an abdominal ultrasound. It has fairly good sensitivity and specificity but it is operator-dependent [2]. Cross-sectional imaging using a CT and/or MRI scan of the abdomen would be the next investigation of choice as it can accurately identify fluid collections while also helping to determine the best approach for management. This can also help differentiate between cystic neoplasm, pseudoaneurysms, duplication cyst, or pancreatic cancers [6]. The main findings of cross-sectional imaging would include a well-circumscribed fluid collection with a well-defined wall that completely encapsulates it.

Endoscopic investigation using EUS is more sensitive compared to cross-sectional imaging in detecting lesions less than 2 cm in diameter. In addition, EUS fine-needle aspiration (EUS-FNA) can help differentiate between benign and malignant cystic lesions, as demonstrated in our case [2]. A further endoscopic modality in the form of ERCP is the gold standard for assessing ductal pathology complicated by pseudocysts. However, due to its invasive nature and high complication rate, MRCP is used instead as the first line for detailed assessment of the biliary system before any intervention.

Double-duct sign, on MRCP and/or CT of the pancreatobiliary system, refers to co-existing dilatation of the CBD and PD. Patients with obstructive jaundice in the context of a double-duct sign have a high incidence of malignancy [7-9]. A pancreatic pseudocyst causing obstructive jaundice with a double-duct sign, as described in our case, is very rare. This is underscored by the paucity of articles encountered on the literature search regarding spontaneous pseudocyst rupture with a double-duct sign.

Pancreatic pseudocysts associated with acute pancreatitis often resolve spontaneously. A period of watchful monitoring with symptomatic conservative management is ideal for uncomplicated cysts. On the other hand, pseudocysts associated with chronic pancreatitis do not usually self-resolve due to extensive damage to the pancreatic duct. Recurrence is not uncommon [10]. Pseudocysts complicated by infection, bleeding, obstructive jaundice, and rupture along with the presence of major symptoms are indications for interventions. Management of complicated pseudocysts can pose considerable challenges for physicians. Often, the treatment requires the involvement of a multidisciplinary team including gastroenterologists, hepatobiliary-pancreatic surgeons, radiologists, and histopathologists. Traditionally, surgery was the first choice of management for complicated pseudocysts, but with the advancement of technology and skills, endoscopic, laparoscopic, and radiologically guided treatments have increasingly taken over.

EUS-guided drainage is now the first choice for complicated pseudocysts [11]. Multiple studies comparing percutaneous drainage and EUS-guided drainage have shown that the latter is superior in terms of greater success rates, less need for re-intervention, less need for re-imaging, lower rate of recurrence after drainage, and shorter hospital stay [12,13].

EUS-guided drainage and surgical drainage have almost equal efficacy and outcome, with similar rates of success and recurrence. EUS is less invasive, less costly, and associated with a shorter hospital stay and more rapid recovery [14-17].

If the pseudocyst communicates with the pancreatic duct, EUS-guided transpapillary drainage becomes the therapy of choice whereas when the pseudocyst causes a visible impression of the gastric or duodenal wall, EUS-guided transmural drainage is the preferred option [18,19]. Our case entailed the gastric transmural approach in order to access the pancreatic pseudocyst. EUS-FNA enabled us to sample the fluid and soft tissue within the pseudocyst, and this was combined with tumor markers to rule out cystic malignancy. This is in accordance with the article published by Vignesh and Brugge, which showed that EUS-guided fluid sampling combined with tumor markers increases the diagnostic yield significantly [20]. The collective data regarding endoscopic drainage show mortality rates of 0-1%, long-term follow-up success rates of 62-75% of cases, and recurrence rates with long-term follow-up of 0-23% [18]. Failure of transpapillary or transmural drainage may make subsequent surgery necessary [19]. Surgical treatment is the preferred method in suspected cystic neoplasia and when complications such as perforation and hemorrhage due to erosion of arteries have occurred [2,20].

## Conclusions

Large pancreatic pseudocysts in the head of the pancreas can compress the CBD and PD, leading to a double-duct sign on cross-sectional imaging. Pancreatic pseudocysts associated with alcoholic chronic pancreatitis can recur with complications like spontaneous rupture and obstructive jaundice. The management of pancreatic pseudocyst can require multiple interventional modalities including EUS-guided drainage, percutaneous drainage, and ERCP.
